# Unmanned aircraft systems as a new source of disturbance for wildlife: A systematic review

**DOI:** 10.1371/journal.pone.0178448

**Published:** 2017-06-21

**Authors:** Margarita Mulero-Pázmány, Susanne Jenni-Eiermann, Nicolas Strebel, Thomas Sattler, Juan José Negro, Zulima Tablado

**Affiliations:** 1 Swiss Ornithological Institute, Sempach, Switzerland; 2 Department of Evolutionary Ecology, Doñana Biological Station, CSIC, Seville, Spain; 3 Departamento de Ciencias Naturales, Universidad Técnica Particular de Loja, San Cayetano Alto, Loja, Ecuador; University of Lleida, SPAIN

## Abstract

The use of small Unmanned Aircraft Systems (UAS; also known as “drones”) for professional and personal-leisure use is increasing enormously. UAS operate at low altitudes (<500 m) and in any terrain, thus they are susceptible to interact with local fauna, generating a new type of anthropogenic disturbance that has not been systematically evaluated. To address this gap, we performed a review of the existent literature about animals’ responses to UAS flights and conducted a pooled analysis of the data to determine the probability and intensity of the disturbance, and to identify the factors influencing animals’ reactions towards the small aircraft. We found that wildlife reactions depended on both the UAS attributes (flight pattern, engine type and size of aircraft) and the characteristics of animals themselves (type of animal, life-history stage and level of aggregation). Target-oriented flight patterns, larger UAS sizes, and fuel-powered (noisier) engines evoked the strongest reactions in wildlife. Animals during the non-breeding period and in large groups were more likely to show behavioral reactions to UAS, and birds are more prone to react than other taxa. We discuss the implications of these results in the context of wildlife disturbance and suggest guidelines for conservationists, users and manufacturers to minimize the impact of UAS. In addition, we propose that the legal framework needs to be adapted so that appropriate actions can be undertaken when wildlife is negatively affected by these emergent practices.

## Introduction

Recently, Unmanned Aircraft Systems (UAS, also known as remote piloted aircraft systems “RPAS” or colloquially as “drones”) have experienced a remarkable development. Although originally UAS were mainly used for military purposes, the technological advances and the miniaturization of components have facilitated the emergence of a mass production industry, which has led to decreasing prices and consequently to a proliferation of the use of UAS in civilian applications. Small UAS (Maximum Take-Off Weight [MTOW] of 250 g—25 kg) are nowadays used for a variety of professional tasks, such as remote sensing, photography, precision agriculture, infrastructures inspection, mining, forestry management, and surveillance, among others [1,2].

Small UAS can provide high spatial and temporal resolution data, which has favored their incorporation to environmental biology in different research and management topics, such as plant and animal ecology [3–6], wildlife monitoring [7,8], anti-poaching [9] and infrastructures impact assessment [10]. There is also a recent increase of operators using UAS to scare off wild birds that cause economic losses in agricultural areas and fisheries, pose risks in airports, damage monuments and interfere in waste management [11–13]. Additionally, UAS are used in ecotourism, hunting, fishing, livestock management and falconry training [14–19]. Small UAS have also become a popular new hobby for the general public for leisure flying and filming, which has resulted in an exponential growth of sales (about 2.5 million units during 2016 in the USA alone;[20]). The expansion on the professional and personal use of small UAS that are operated over any terrain, frequently in natural areas, and at low altitudes above ground level (AGL < 500 m), makes the systems susceptible to interact with the local fauna, constituting a new potential source of anthropogenic disturbance.

There is abundant literature about the effects of disturbance on wildlife. Animal responses to on-foot human presence [21], terrestrial vehicles [22–27] and aerial platforms such as manned aircraft, helicopters and gliders [28–30] may vary from punctual behavioral or physiological reactions to reductions in fitness (e.g. mortality by collision with the vehicles or stress-related decrease in productivity) or changes in spatial use (e.g. avoidance of certain areas; [21]) that may fragment or compromise the viability of populations. The studies indicate that the factors determining the probability and intensity of animal responses depend on the one hand on the characteristics of the disturbing agent (e.g. size, noise emitted, speed, distance, angle of approach), which affect the perception of the risk by the animal [21,31]. The larger and noisier the approaching agent is, the stronger the anti-predator responses will be [32]. Animals have also been shown to react at larger distances when the approach is faster and more directional towards them [33,34, but see also 35]. On the other hand, animal responses also depend on the characteristics and context of the animal exposed to the disturbance (e.g. species, age, level of aggregation, life history stage, habitat, season), which explain the susceptibility of the animal to react [21,31]. Different types of animals show different reactions to disturbance. Some species are more prone to conduct flight and fight responses, while others seek for protection by forming large aggregations, hiding in vegetation or using crypticity strategies, and do not show observable responses despite being physiologically stressed [36–38]. Similarly, reproductive status can also condition the animals’ response, since gravid females might have reduced moving capabilities, and individuals involved in parental care may prioritize protection of their offspring to their own survival [39,40].

Due to the recent expansion of UAS use, assessment of their impact is currently restricted to isolated [41,42] or descriptive studies [6,43,44] and lacks a broad scientific base. The aim of our study is to identify the factors influencing the probability and magnitude of wildlife reactions to UAS. We reviewed the literature searching for information about animal responses to small UAS and then performed a combined analysis of the resulting database. According to the existing studies about disturbance, we hypothesized that the response of wildlife to UAS would depend on both the properties (aircraft size and engine) and flight pattern of the aircraft, and the attributes and context of the animals being concerned (animal type, life-history stage and level of aggregation). Finally, we discuss the implications of our findings in the context of human-disturbance ecology and species conservation and provide recommendations to guarantee safe operation while minimizing disturbance, which might be of interest to conservationists and managers of protected areas, as well as to UAS manufacturers, users, and regulators who require solid baseline information for or against UAS use in specific contexts.

## Material and methods

We performed a comprehensive literature search up to December 31^st^, 2015. One person performed the searches in English and Spanish and another person did the search in German with the same criteria. On a consensual way, we gathered information from scientific articles, dissertations, and reports mentioning the use of small UAS in animal monitoring and ecology related topics. In the search we applied the keywords: ‘unmanned aircraft/aerial system’; ‘remote piloted aircraft system’; ‘UAS’; ‘UAV’; ‘drone’; ‘RPAS’; ‘radio control aircraft’; ‘model aircraft’ and variations of these terms, combined with: ‘wildlife’; ‘survey’; ‘inventory’; ‘impact’; ‘monitoring’ and similar ones. Searches were made mainly using databases (Google Scholar, Scopus and Web of Science) but also complemented through reference harvesting, citation tracking, abstracts in conference programs, and author search (see, for example [45,46]). Studies conducted using radio-controlled aircraft (RC hereinafter) were also included in this review because they have the same physical characteristics as UAS, and therefore may be equivalent in terms of impact on wildlife. In contrast, we excluded information referring to large UAS (MTOW>25 kg) because their operation takes place at high altitudes, thus their impact may be comparable to the effects produced by manned aircraft. Similarly, we excluded references to UAS with a MTOW below 250 g, since these are normally operated within short ranges (a few meters) around the pilots and often indoors, all of which makes them less susceptible to interfere significantly with wildlife. The search provided a total of 82 records, from which we retained 54 publications that indicated actual UAS or RC flights performed over wildlife in fieldwork. From these, we only used the ones explicitly indicating whether small UAS did or did not affect wildlife (i.e. 36 publications). The resulting database was completed with our own unpublished data from 17 UAS field campaigns (250 flights) conducted from 2011 to 2014 (for a PRISMA Flowchart see S1 Checklist and S1 Text, for unpublished campaigns see S2 Text, for a complete list of studies see S3 Text). All the field procedures were reviewed and accepted by the authority yielding the permit of use (Doñana National Park authorities, permits for Aeromab P07-RNM-03246 and Planet GA 257649 projects, Junta de Andalucía). Data on semi-captive animals, nestlings, roosting birds, or nocturnal UAS flights were omitted from subsequent analyses due to the low number of records and the lack of comparability of responses. The physiological responses and the long-term consequences of the flights on animals could not be further explored due to insufficient evidence.

From retained studies we collected all available information on animals' reaction to UAS. To investigate the determinants of these reactions, we also recorded any characteristics of the flight, aircraft, or the animals concerned, which could be influencing animal responses according to previous literature (see for example [21,42]). Unfortunately, many of these variables could not be included in further analyses due to incomplete information (e.g. habitat type) or correlation with other variables (e.g. aircraft shape is correlated with flight pattern and aircraft size).

In order to standarize the animal behaviors described in the different studies, we classified the *reaction* caused by the UAS into the following categories: “none” (i.e. study mentioned no noticeable behavioral change), “alert reaction” (i.e. at least one individual showed increased attention or alert towards the UAS during the study), or “active reaction” (i.e. at least one individual responded actively towards the UAS, either fleeing or attacking).

UAS-dependent parameters that we were able to record from the publications (or from technical data-sheets of the UAS manufacturer) were the characteristics of the flight, the *size* of the aircraft (i.e. largest measure of the vehicle in any dimension), and the *engine type* as a proxy of noise (i.e. “fuel” being noisier than “electric”). The flight was characterized by the *flight pattern* followed in the study and by the spatial relation of the aircraft to the animals when the reaction, or lack of it, was observed.

The *flight pattern* was summarized in three categories: “target-oriented”, for those that approached the animal either directly or in consecutive vertical-horizontal approaching steps, “lawn-mower”, entailing a flight with systematic turns back and forth at a constant AGL to scan an area, and “hobby”, which included the acrobatic or irregular flight patterns (e.g. leisure flights) typically performed by RC planes. The spatial relation of the aircraft to the animals was measured either by the UAS altitude above ground level *AGL* (in meters) or by the *distance* (in meters) to the UAS/RC, depending on what was provided on each study. When a given reaction was mentioned in a study (i.e. “alert response” or “active response” of at least one individual), we recorded the maximum AGL or distance, depending on availability, at which the reaction was observed as the threshold at which the effect started. When the AGL or distance at which the reactions occurred was not given, but they only provided the range of values at which the UAS was flown, we retained consistently the lowest/shortest value to avoid overestimation of the impact. When no reaction (“none”) was observed, we took the lowest/shortest value provided in order to represent how close fauna was approached without causing any disturbance.

As for the characteristics of the animals subjected to UAS flights, we recorded the *species* (i.e. lowest taxonomic level available), which was later classified in five *animal types* according to the expected differences in response to UAS: “flightless birds” (i.e. penguins), “large birds” (flying birds with average body size longer than 70 cm and heavier than 1 kg), “small birds” (flying birds smaller than the previous category), “terrestrial mammals” (mammals being on terrestrial habitats during the flights: carnivores, pinnipeds, primates and ungulates), and “underwater species” (i.e. vertebrate species permanently underwater, such as fish or cetaceans) (for a complete list of species and studies see S3 Text). We also gathered additional information on the *life history stage* and *level of aggregation* of the animals. *Life history stage* was defined as “breeding” if the species were known to be within the period of gestation or parental care in the moment in which flights were performed, and “non-breeding” for cases in which species were outside their gestation or parenting season. The *level of aggregation* was classified as “solitary” individuals, “small group” (2 to 10 individuals), “medium group” (11 to 30 individuals), and “large group” (more than 30 individuals). Animal characteristics not directly provided in the publication were approximated from specialized sources, such as handbooks or field guides (e.g. the Handbooks of the Birds of the World [47]).

### Statistical analysis

In order to determine which of the factors recorded are affecting animal responses to UAS, we selected a sequential analysis approach that allowed us to integrate information from all studies, which varied importantly in the type of information given, and to cope with correlated explanatory variables (e.g. *AGL* and *flight pattern*).

In a first step we pooled all data to examine which factors determined the impact of UAS. For this, we fitted two different logistic mixed models with *reaction* as the response variable. In the first model, we only distinguished between reaction (“1”, when “alert” or “active” response was observed) and absence of reaction (“0”, “none”). In the second model we focused on the intensity of the reaction. For that, we only considered studies where a reaction was observed. The dependent variable was set to “0” when the response was weak (“alert”) and “1” when the reaction was strong (“active” reaction including attack and fleeing). In both models, we tested for the effect of *flight pattern*, *engine type*, and *animal type* as explanatory variables. Further variables could not be included due to either correlation issues or missing values.

In a second step we focused only on the cases showing active responses (mostly fleeing) and used mixed models to examine at which *AGL* or *distance* from the UAS these responses occurred. Note that the AGL and distances used here are conservative and represent the AGL or distances at which some animals start to actively react, but not necessarily the ones at which all observed animals react. We analyzed the data for each *flight pattern* separately and tested the effect of variables that we could not investigate in the first step. Note that most data in this second-step analyses are referred to birds, except for seven cases of terrestrial mammals for the “target-oriented” and one for the “lawn-mower” pattern. To analyze the data on “target-oriented” and “lawn-mower” patterns respectively, we used the log-transformed *AGL* as the response variable, which followed a normal distribution. As explanatory variables, we tested for the effect of *life history stage* and the *size of the UAS*. Given that sample size was larger for the “lawn-mower” case, we could also include the *engine type*. For the “hobby” pattern data, usually *distance* to animals was provided instead of *AGL*. Thus, we used the log-transformed *distance* as dependent variable, also following a normal distribution, and were able to test for the effect of *life history stage* and the *level of aggregation* as explanatory variables.

In all five mixed models we included three random factors to account for the autocorrelation among data within the same study (*reference*), same species (*species*), and same family (*family*). All models were performed with the software R version 2.15.1 [48] using the “lmer” and “glmer” functions of the package lme4, for normal and binomial distributions respectively [49]. We used the Bayesian framework to obtain the credible intervals (CrI) of the estimated effects. For this, we simulated a random sample (N = 5000) from the joint posterior distribution of the model parameters using the function “sim” of the package arm [50]. The 2.5% and 97.5% quantiles from this sample were then used as the lower and upper limits of the 95% CrI and the 5.0% and 95.0% quantiles as the extremes of the 90% CrI. An effect was considered to reach standard significance levels when its 95% CrI did not contain zero and near-significance levels when 90% CrI did not contain zero (S1 and S2 Tables).

## Results

**Fig 1:**
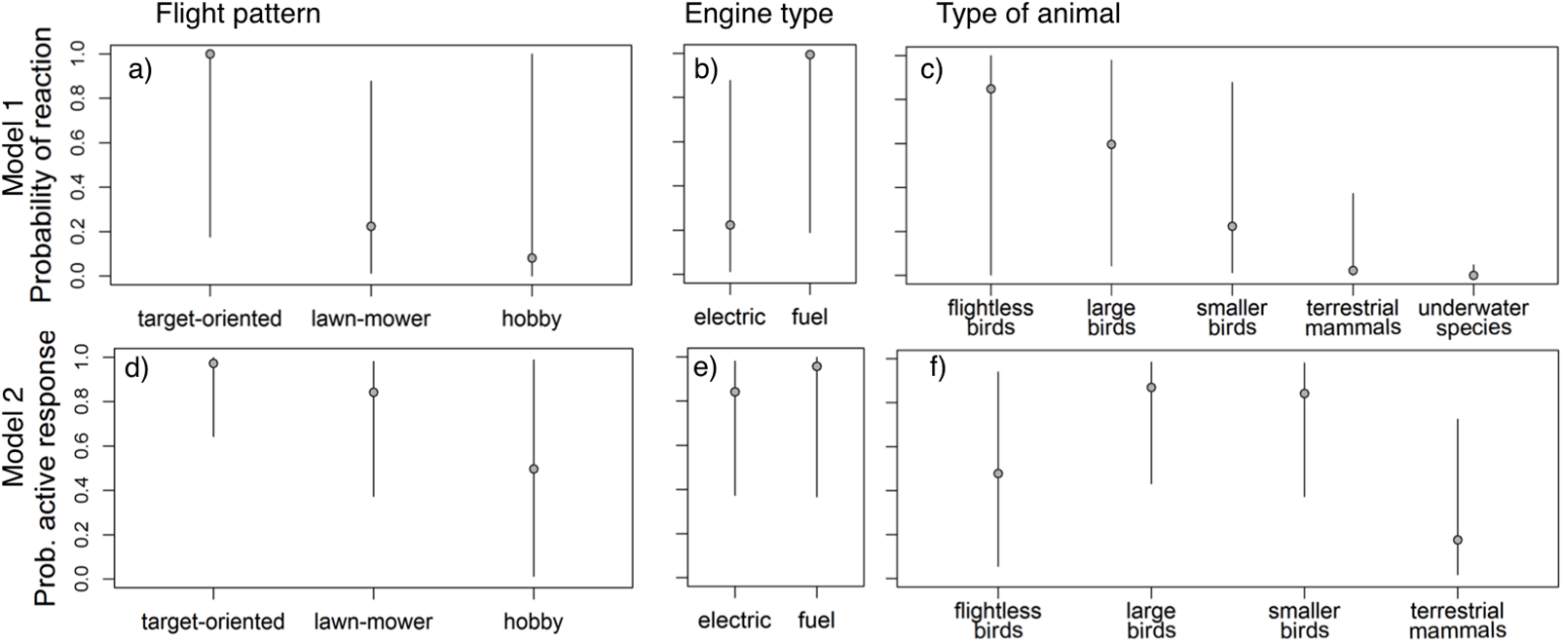
Mean probability of wildlife reaction with 95% CrI (S1 Table) according to UAS flight pattern (a, d), UAS engine type (b, e), and type of animals concerned (c, f). (Model 1: 0 = no reaction vs. 1 = reaction [either alert or active; N = 167]; Model 2: 0 = alert vs. 1 = active response; N = 106).

Overall, we were able to include in the analyses data from 36 published studies and from 17 unpublished field campaigns (S3 Text) that reported information about wildlife reactions towards UAS flights. The first-step analyses (i.e. pooled data) showed that the probability of UAS producing a reaction on wildlife was higher in studies conducting “target-oriented” than “lawn-mower” flight patterns (standard significance levels were not reached but the 90% CrI did not include zero; Fig 1a; S1 Table). The effect of the “hobby” pattern, however, was not clearly different from any of the other two patterns. A similar but less strong trend was found when we compared the intensity of the response (“alert” vs. “active” escape or attack), with “target-oriented” flight patterns being more likely to cause an active response than the “lawn-mower” flights (Fig 1d; S1 Table). UAS with fuel engines also increase (according to the 90% CrI) the likelihood to produce animal reactions than electric systems (Fig 1b; S1 Table). There are also significant differences (i.e. 95% CrI not including zero) among species regarding the response to UAS (Fig 1c and 1f; S1 Table). The less responsive species were the ones living underwater, for which we only found one record mentioning a reaction, followed by terrestrial mammals. The groups that showed the highest sensitivity to UAS were birds, and within birds, the overall probability of responding tended to be higher in flightless and large flying birds than in smaller flying birds, while the intensity of the response was higher in both flying-bird groups than in the flightless ones. However these within-bird trends did not reach significance levels (according neither to the 95% CrI nor the 90% CrI).

**Fig 2:**
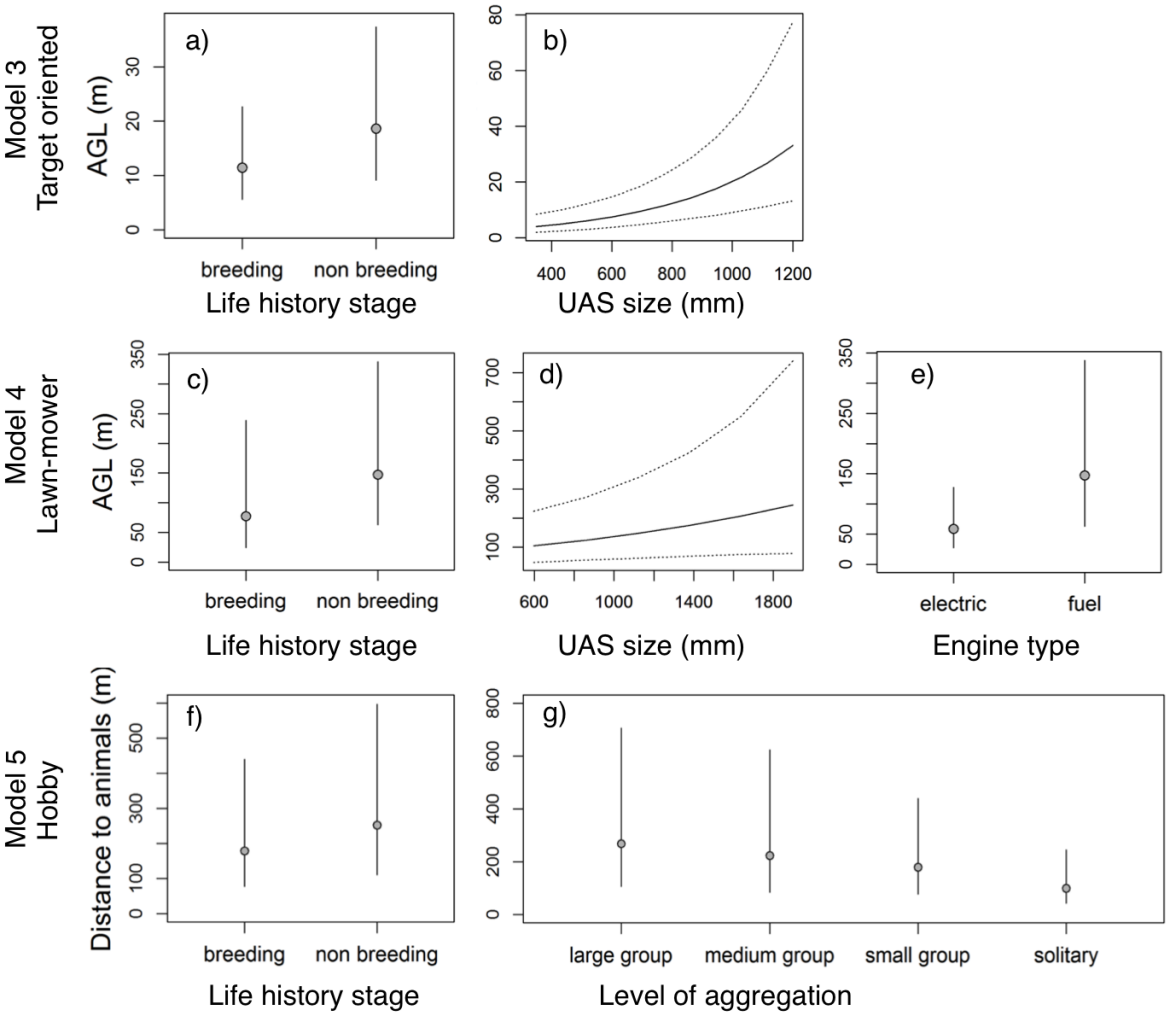
Factors influencing the AGL (m) with 95% CrI (S2 Table) at which animal respond actively during target-oriented flights (Model 3; N = 12; a) life-history stage and b) UAS size), and during lawn-mower pattern flights (Model 4; N = 27; c) life-history stage, d) UAS size, and e) engine type). Distance (m) with 95% CrI (S2 Table) at which animals show an active response during hobby (Radio-controlled vehicles) flights (Model 5; N = 21; f) life-history stage and g) level of aggregation).

In the second-step analyses, examining active animal responses, we found that animal-specific characteristics, such as *life-history stage* and *level of aggregation*, were important to determine how close individuals can be approached without causing them to respond actively (usually fleeing). For all three types of flight patterns, individuals outside the gestation or parental-care periods responded at significant (or near-significant) larger AGL (i.e. vertical distance) or distances from the UAS than animals in those breeding stages (Fig 2a, 2c and 2f; S2 Table). Individuals also fled at significantly longer distances when they were in larger groups than when they were in small groups or solitary (Fig 2g; S2 Table). The results also demonstrated an influence of the size of UAS, with larger (within the studied range) aircraft causing active reactions at higher AGL than smaller ones (Fig 2b and 2d; S2 Table).

## Discussion

Our findings support our hypothesis that animal reactions to UAS are conditioned by factors related to both UAS attributes and mode of operation, and also to the characteristics of the concerned animals. The flight pattern of the UAS has an important effect on wildlife responses. Target-oriented flights, which are conducted towards the focal animals and usually approach them at shorter AGL and distances, mainly for photography, nest inspections, and recently for “animal control” (e.g. [11]), produce more reactions than the other flying patterns, leading to higher disturbance. On the other extreme, lawn-mower flights, generally conducted for mapping, surveillance and wildlife census, performed at higher altitudes and following regular trajectories are less likely to affect the animals. This result agrees with studies on manned aircraft showing that directness of aircraft approach influence animal responses [29,51,52] and could be related to anti-predator behavior, since animals perceive higher risks when the threat is on a trajectory towards them [53].

We detected that fuel engines (e.g. [54]), generally noisier than electric ones, produce more animal reactions, which would agree with previous authors that indicate the importance of noise as a source of disturbance for wildlife [55]. Besides, it has been suggested that animal reactions are not only influenced by the noise level itself but also by changes on noise intensity. In the case of a UAS these changes in intensity may be associated with aircraft on-flight engine variations due to speed or trajectory changes or wind alterations [41,56]. On the other hand, silent UAS platforms or UAS operated at high altitudes (e.g. over 100 m AGL) where noise is attenuated, may be hard to hear by wildlife and therefore less distressing [57–59]. The size of the UAS also affects animal reactions, with larger platforms producing responses at larger AGL than small ones, probably because the size of the threat increases perceived risk [53] and the probability of detecting it.

As hypothesized, we found substantial differences in occurrence of response to UAS among different animal types. Birds are the most sensitive to UAS, with flightless birds and large birds being more likely to show reactions than smaller ones, probably due to a higher range of detection for larger size species [60]. Terrestrial mammals are overall less reactive to UAS than birds. We found that fully aquatic animals are the least affected animal type, in line with a recent review [43], which could be explained by the water layer providing some isolation from aerial stimuli. The differences detected between animal types in their response to UAS can be also related to their anti-predatory strategies, with species that are naturally threatened by aerial predators (e.g. birds) reacting more than other animal types, such as large terrestrial mammals and fully aquatic species.

We found that animal-specific characteristics, such as life-history stage and level of aggregation, have an influence on the animals’ response. We found that breeding animals (gravid or providing parental care, in our case mostly breeding birds) are on average less inclined to flee than non-breeders, probably due to a reluctance to abandon the progeny (i.e. nest) or to movement difficulties for gravid females [21,31]. In some cases, however, reproducting individuals can also react aggressively to UAS [29,61], probably because of an increase in territoriality during this period or defense of the progeny. As for the level of aggregation, our results showed that animal group size increases active reactions towards UAS, in line with observations towards manned aircrafts [42,56] and suggested for UAS [42].

We acknowledge that there might be other important factors affecting animal responses to UAS that we could not explore quantitatively in this study due to incomplete information or correlation with other variables. For example, Vas et al. [42] and Rümmler et al. [62] indicate that UAS vertical approaches had a higher impact than horizontal ones, and [42] indicate that it could be because birds associate the approach with a predator attack, while Rümmler et al. [62] suggest that the noise emission in this direction may be higher. The shape of the UAS could also have an influence on its disturbance, as predator-like shapes seem to produce more reactions in birds [63], but more standardized experiments should be performed to fully assess the effect of this variable. The time of the day might also play a role, since at night or dusk animals tend to react with less intensity [64]. From the field of manned-aircraft there is evidence that habitat characteristics and animal behavior before the flight affect their reactions. That is, open habitats favor fleeing responses compared to closed habitats [29] and animals involved in active behaviors are more prone to react to disturbances than passive ones [65].

Currently we have insufficient information on the physiological and long-term consequences of UAS disturbances. However, there are some indications that UAS might cause non-visible effects. For example, Ditmer et al. [41] found increases in physiological stress but not behavioral changes in animals subjected to close-distance UAS approaches, as has been also shown in on-foot disturbances [38]. Long-term studies on the use of UAS have not yet been performed; however, according to human disturbance literature (see [21] for a comprehensive review), the physiological or behavioral stress potentially caused by disturbance may lead to higher energy expenditures, decreases in reproduction and survival, and space-use changes, which might compromise the average fitness or even viability of certain populations. In agreement to this, studies conducted on RC fields indicate that abundant flights may lead to territory abandonment and decreased productivity in sensitive bird species [56,66–68] or to habituation in less sensitive ones [56,69]. We agree on the need of further research (with standardized well-thought study designs) to increase our knowledge on the effect of other factors and on the potential consequences for individuals and populations. However, we believe that the generalized patterns shown here, despite the variability in their design and purpose of the reviewed studies, are a good first step towards a better understanding of the effects of using UAS and the ways to mitigate their impact.

## Conclusions and management implications

UAS constitute a potential new source of anthropogenic disturbance, and the extent to which they elicit wildlife reactions depends on UAS and animal related factors. Smaller UAS, electric engines, and lawn-mower flight patterns generally evoke no disturbance at all or only a short disturbance, the latter comparable to those induced by natural predators. More intense reactions are observed in bird species, larger group sizes, and animals in non-breeding stage. AGL or distances at which animals were observed to flee during UAS flights are smaller (usually below 500 m) than for manned aircraft (105 m-15 km [28,70]) and for car approaches (e.g. for ungulates, 50 m—2.8 km [71]), and are comparable to those reported for on-foot approaches (e.g. for birds: 0–455 m in [72,73] respectively). UAS also produce less noise and have a smaller size than manned aircraft or cars (see S4 Text for car vs. UAS noise comparison), when flying at average engine power. Hence, the careful use of UAS might be a valuable alternative for methods such as bio-logging, on-foot, and manned aircraft censuses used in biological studies. In contrast, when UAS perform direct and too close approaches to animals or sensitive structures such as nests, they can evoke more disturbances to wildlife. Therefore, we recommend that UAS flights are avoided unless they constitute the least invasive option for necessary wildlife studies, and discouraged if they are performed just for leisure purposes such as flying or filming. As UAS hobby uses are on the rise, some authorities have established regulatory restrictions to prevent undesirable consequences for fauna based on the precautionary principle. Along this line, the use of UAS has been banned in the United States National Parks [74], in reserves for waterbirds and migratory birds in Switzerland [75] and in London green spaces [76], and some drone pilots have received fines for flying in protected areas [77,78].

We recommend using UAS only in projects where flights are justified and adopting the following guidelines (provided with more detail in S5 Text and that can be complemented with the ones suggested by [44]) that are made on the basis of the quantitative analysis we presented here, the qualitative information extracted from the literature reviewed and own experience on UAS flights over wildlife,: 1) Use reliable UAS operated by experienced pilots; 2) Favor low-noise or small UAS against noisier or larger ones; 3) Mount the ground control station 100–300 m away from the study area; 4) Conduct missions as short as possible; 5) Fly at the highest altitude possible; 6) Avoid maneuvers above the animals; 7) Favor lawn-mower flight patterns; 8) Minimize flights over sensitive species or during breeding period; 9) Avoid UAS silhouettes that resemble predator shapes; 10) Avoid close-distance direct approaches and favor indirect ones; 11) Monitor target animals before, during, and after the flight; 12) For nest inspections, fly at times in which eggs/chicks are out of risk; 13) If the flights are around aggressive raptor’s territories, perform them at day times when the temperature is low and birds are less prone to fly.

These recommendations should be updated when more information is available because, as revealed here, there are many factors that condition the impact of UAS missions. There is an urgent need for providing the appropriate information to UAS users and authorities to minimize the impact that these systems can produce on wildlife. This can be done with dissemination campaigns, offering guidelines with the products and cooperating with manufacturers. It would also be advisable to adapt the legal frame so that appropriate actions can be undertaken when wildlife is negatively affected by these emergent practices, specifying drone use on the animal welfare legislation, or including references to wildlife impact on the framework for UAS in the civil aviation regulations.

## Supporting information

S1 Text PRISMA 2009 checklist. (DOC)

S2 Text Methodology for UAS field campaigns (unpublished data). (DOCX)

S3 Text List of studies/cases used in the analyses. (DOCX)

S4 Text UAS and car sound comparison. (DOCX)

S5 Text Guidelines to minimize UAS impact on wildlife. (DOCX)

S1 Table Results of the GLMMs exploring the factors affecting the probability of observing a reaction in wildlife exposed to UAS flights (Model 1) and the determinants of the probability of observing an active vs. a passive response to the UAS (Model 2). (DOCX)

S2 Table Results of the GLMMs investigating the factors determining the AGL (Model 3 and 4) or distance to the fauna (Model 5) at which UAS were when an active response (mostly escape) was observed. (DOCX)

S1 Checklist PRISMA flowchart. (PDF)
