# Supplementary material for: Unmanned aircraft systems as a new source of disturbance for wildlife: A systematic review
Source: PLoS One. 2017 Jun 21;12(6):e0178448. doi: 10.1371/journal.pone.0178448 (PMC5479521; doi:10.1371/journal.pone.0178448)
Supplement: S2 Text — (DOCX) [file pone.0178448.s002.docx]

**S2 Text. Methodology for UAS field campaigns (unpublished data)**

From 2011 to 2014 a team from Doñana Biological Station, Spanish Council for Scientific Research (CSIC) performed 17 UAS field campaigns in the frame of Aeromab and Planet research projects, conducting about 250 flights. Most of the field campaigns took place in Doñana National Park (South West of Spain), a protected area mainly composed by Mediterranean forest and marshlands, two campaigns were performed in agricultural fields around Seville (Spain) and one in different locations of South Africa with savanna and forest habitats. Twelve of these UAS campaigns were aimed to bird monitoring and five to mammal research. All campaigns were conducted with electric small fixed wing UAS: 16 with a modified Easy Fly St-330 (for details see Mulero-Pázmány et al. 2014b), and one with the Elimco Viewer E300 (http://www.elimco.com/p_UAV-E300_24.html).

The operative protocol for UAS operations was similar in all the cases, with the ground control station placed 500 m or further from the target species, from where the UAS took off and gained altitude (10-400 m AGL). The flights were conducted over the animals, following a “mower” pattern covering the area to overfly, without abrupt changes of speed and trajectory unless there was any technical emergency and had an average duration of 32 minutes. The field campaigns complied with the current Spanish and South African legislation involving aviation safety and field technicians had the required licenses to operate in the frequencies used for the work.
