# Supplementary material for: Unmanned aircraft systems as a new source of disturbance for wildlife: A systematic review
Source: PLoS One. 2017 Jun 21;12(6):e0178448. doi: 10.1371/journal.pone.0178448 (PMC5479521; doi:10.1371/journal.pone.0178448)
Supplement: S3 Text — (DOCX) [file pone.0178448.s003.docx]

**S3 Text. List of studies/cases used in the analyses**

| **Reference** | **Species** | **UAS type** | **Area, country** |
| --- | --- | --- | --- |
| Acevedo-Whitehouse *et al.* 2010 | *Balaenoptera edeni, Balaenoptera musculus, Balaenoptera physalus, Delphinus sp, Eschrichtius robustus, Megaptera novaeangliae, Physeter macrocephalus, Tursiops truncatus* | Rotary wing | Gulf of California, Pacific coast of Baja California, Mexico |
| Barasona *et al.* 2014 | *Bos taurus, Cervus elaphus, Dama dama, Equus caballus, Sus scrofa* | Fixed wing | Doñana NP, Spain |
| Bivings 1988 | *Anatidae, Anserinae, Cathartidae, Charadriidae, Icteridae, Passer domesticus, Scolopacidae* | Fixed wing | USA |
| Boscher 1993 | *Numenius arquata* | Fixed wing | Rhine Valley, Baden-Württemberg, S Germany |
| Chabot and Bird 2012 | *Branta canadensis, Chen caerulescens* | Fixed wing | Ste-Anne-de-Bellevue, QC, Baie-du-Febvre, QC, Canada |
| Chabot *et al.* 2015 | *Sterna hirundo* | Fixed wing | Kouchibouguac National Park, New Brunswick, Canada |
| Cunningham 2014 | *Eumetopias jubatus* | Rotary wing | Alaska, USA |
| Degollada *et al.* 2012 | *Cetaceans* | Fixed wing | Spain |
| Dietrich *et al.* 1989 | *Anas platyrhynchos, Ardea cinerea, Limosa limosa, Vanellus vanellus* | Fixed wing | Grother Mersch, Quakenbrück, Osnabrück, Germany |
| Ditmer *et al.* 2015 | *Ursus americanus* | Rotary wing | Northwestern Minnesota, USA |
| Dulava *et al.* 2015 | *Aechmophorus clarkia, Aechmophorus occidentalis, Anas carolinensis, Anas clypeata, Anas platyrhynchos, Aythya collaris, Bucephala albeola, Cygnus columbianus, Larus occidentalis, Melanitta perspicillata, Oxyura jamaicensis, Phalacrocorax auritus* | Rotary wing | Tomales Bay, Ruby Lake NWR, USA |
| Durban *et al.* 2015 | *Orcinus orca* | Rotary wing | Vancouver Island, British Columbia, Canada |
| Gardner *et al.* 2010 | *Arctocephalus gazelle, Pygoscelis antarctica, Pygoscelis papua* | Rotary wing | South Shetland Islands.Admiralty Bay, King George Island and Cape Shirreff on Livingston Island, Antarctica |
| Goebel *et al.* 2015 | *Arctocephalus gazelle, Hydrurga leptonyx, Leptonychotes weddellii, Pygoscelis antarctica, Pygoscelis papua* | Rotary wing | Cape Shirreff, Livingston Island, South Shetland Islands, Antarctica |
| Grenzdörffer 2013 | *Larus canus* | Rotary wing | Langenwerder island, Germany |
| Ivoševic *et al.* 2015 | *Anatidae* | Rotary wing | Chiaksan National Park, Taeanhaean National Park, South Korea |
| Jones *et al.* 2006 | *Bos taurus, Eudocimus albus, Mycteria americana, Trichechus manatus latirostris* | Fixed wing | Florida, USA |
| Junda *et al.* 2015 | *Pandion haliaetus* | Rotary wing | Canada and USA |
| Komenda-Zehnder and Zehnder 2010 | *Aquila chrysaetos* | Fixed wing | Toggenburg, Kanton Sankt Gallen, Switzerland |
| Koski *et al.* 2015 | *Balaena mysticetus* | Fixed wing | Igloolik, Nunavut, Canada |
| Kudo *et al.* 2012 | *Oncorhynchus keta* | Rotary wing | Moheji River, Southern Hokkaido, Japan |
| Liu *et al.* 2015 | *Platalea minor* | Fixed wing | Taijiang National Park, China |
| Moreland *et al.* 2015 | *Histriophoca fasciata*  *Phoca largha* | Fixed wing | Bering sea ice edge |
| Mulero-Pázmány et al. unpublished-Bela Bela 2012 | *Aepyceros melampus, Damaliscus pygargus, Hippotragus niger, Tragelaphus strepsiceros* | Fixed wing | Bela Bela, South Africa |
| Mulero-Pázmány et al. unpublished-Seville 2011-2012 | *Circus pygargus* | Fixed wing | Seville, Spain |
| Mulero-Pázmány et al. Doñana NP. unpublished-2009-2013 | *Anas acuta, Anas clypeata, Anas platyrhynchos, Anser anser, Ardea cinerea, Aythya ferina, Bubulcus ibis, Bos taurus, Cervus elaphus, Ciconia ciconia, Dama dama, Ardea alba, Egretta garzetta, Equus caballus, Fulica atra, Himantopus himantopus, Larus genei, Netta rufina, Phoenicopterus roseus, Platalea leucorodia, Plegadis falcinellus, Podiceps cristatus, Porphyrio porphyrio, Sus scrofa, Tachybaptus ruficollis* | Fixed wing | Doñana NP, Spain |
| Mulero-Pázmány *et al.* 2014 | *Ciconia ciconia* | Fixed wing | Doñana NP, Spain |
| Mulero-Pázmány *et al.* 2014b | *Equus quagga, Giraffa camelopardalis, Rhinocerotidae* | Fixed wing | KwaZulú-Natal, South Africa |
| Pomeroy *et al.* 2015 | *Halichoerus grypus*  *Phoca vitulina* | Rotary wing | Abertay Sands, Isle of May, North Rona, Loch fleet, Scotland |
| Potapov *et al.* 2013 | *Haliaaetus lagicus* | Rotary wing | Magadan State Reserve, Russia |
| Putzer 1989 | *Anatidae, Ardea cinerea, Aythya ferina, Aythya fuligula, Bucephala clangula, Callidris alpine, Charadrius dubius, Gallinago gallinago, Haematopus ostralegus, Phalacrocorax carbo, Mergus merganser, Pluvialis apicaria, Pluvialis squatarola, Vanellus vanellus* | Fixed wing | France and Germany |
| Ranftl 1990 | *Motacilla flava* | Fixed wing | Germany |
| Ratcliffe *et al.* 2015 | *Aptenodytes patagonicus*  *Pygoscelis papua* | Rotary wing | East Falkland, UK |
| Rümmler *et al.* 2015 | *Pygoscelis adeliae*  *Pygoscelis papua* | Rotary wing | King George Island, Antarctica |
| Rümmler 2015 unpublished | *Arctocephalus gazelle, Leptonychotes weddellii, Macronectes giganteus, Mirounga leonina* | Rotary wing | Antarctica |
| Sweeney *et al.* 2015 | *Eumetopias jubatus* | Rotary wing | Alaska, USA |
| USGS 2011 | *Grus canadensis* | Fixed wing | Monte Vista National Wildlife Refuge, Colorado, USA |
| Vas *et al.* 2015 | *Phoenicopterus roseus, Tringa nebularia* | Rotary wing | Zoo du Lunaret, Montpellier,  Etang de l’Or, Candillargues, France |
| Vermeulen *et al.* 2013 | *Kobus kob kob, Loxodonta africana, Papio anubis* | Fixed wing | Nazinga Game Ranch, Burkina Faso |
| Weissensteiner *et al.* 2015 | *Corvus [corone] cornix* | Rotary wing | Uppsala län, Sweden |

**References**

Acevedo-Whitehouse K, Rocha-Gosselin A, and Gendron D. 2010. A novel non-invasive tool for disease surveillance of free-ranging whales and its relevance to conservation programs. *Anim Conserv* **13**: 217–25.

Barasona JA, Mulero-Pázmány M, Acevedo P, *et al.* 2014. Unmanned Aircraft Systems for studying spatial abundance of ungulates: relevance to spatial epidemiology. *PLoS One* **9**: e115608.

Bivings AE. 1988. Advantages and limitations of Radio-Controller aircraft in bird dispersal. In: Bird Strike Committee Europe 19. Madrid, Spain.

Boscher M. 1993. Auswirkungen von Modellflug und Straßenverkehr auf die Raumnutzung beim Grossen Brachvogel (*Numenius arquata*). *Z Ökologie u Naturschutz* **2**: 11–8.

Chabot D and Bird DM. 2012. Evaluation of an off-the-shelf Unmanned Aircraft System for surveying flocks of geese. *Waterbirds* **35**: 170–4.

Chabot D, Craik SR, and Bird DM. 2015. Population census of a large common tern colony with a Small Unmanned Aircraft. *PLoS One* **10**: 1–14.

Cunningham KW. 2014. Augmenting steller sea lion surveys in the Western Aleutians with Unmanned Aircraft. – Project number 1120. Fairbanks AK.

Degollada E, Amigó N, Bou M, *et al.* 2012. A novel technique for cetacean localization and surveillance by means of a radio-controlled aircraft. In: 26th Conference of the European Cetacean Society. ECS, Galway, Ireland.

Dietrich K, Koepff C, Mühlen G von der, and Steiof K. 1989. Untersuchungen über die Auswirkung von Modellflugbetrieb auf das Verhalten von Wiesenvögeln. *Unveröffentlichtes Gutachten*: S76.

Ditmer MA, Vincent JB, Werden LK, *et al.* 2015. Bears show a physiological but limited behavioral response to Unmanned Aerial Vehicles. *Curr Biol* **25**: 2278–83.

Dulava S, Bean WT, and Richmond OMW. 2015. Applications of Unmanned Aircraft Systems (UAS) for waterbird surveys. *Environ Pract* **17**: 201–10.

Durban JW, Fearnbach H, Perryman WL, and Leroi DJ. 2015. Photogrammetry of killer whales using a small hexacopter launched at sea. *J Unmanned Veh Syst* **3**: 1–5.

Gardner S, LeRoi D, and Perryman W. 2010. A penguin population polar express: NOAA’s quest to count penguin breeds speeds up with a VTOL UAS. *Unmanned Syst* **29**: 30–5.

Goebel ME, Perryman WL, Hinke JT, *et al.* 2015. A small unmanned aerial system for estimating abundance and size of Antarctic predators. *Polar Biol*.

Grenzdörffer GJ. 2013. UAS-based automatic bird count of a common gull colony. In: Int. Arch. Photogramm. Remote Sens. Spatial Inf. Sci., XL-1/W2. Rostock, Germany.

Ivoševic B, Han Y-G, Cho Y, and Kwon O. 2015. The use of conservation drones in ecology and wildlife research. *J Ecol Environ* **38**: 113–8.

Jones G, Pearlstine L, and Percival H. 2006. An assessment of small unmanned aerial vehicles for wildlife research. *Wildl Soc Bull* **34**: 750–8.

Junda J, Greene E, and Bird DM. 2015. Proper flight technique for using a small rotary- winged drone aircraft to safely, quickly, and accurately survey raptor nests. *J Unmanned Veh Syst* **15**: 1–15.

Komenda-Zehnder S and Zehnder M. 2010. Kurzbeiträge Angriff eines Steinadlers Aquila chrysaetos auf ein Modellsegelflugzeug. *Der Ornithol Beobachter* **107**: 111–3.

Koski WR, Gamage G, Davis AR, *et al.* 2015. Evaluation of UAS for photographic re-identification of bowhead whales, *Balaena mysticetus*. *J Unmanned Veh Syst* **3**: 22–9.

Kudo H, Koshino Y, Eto A, *et al.* 2012. Cost-effective accurate estimates of adult chum salmon, *Oncorhynchus keta*, abundance in a Japanese river using a radio-controlled helicopter. *Fish Res* **119-120**: 94–8.

Liu C-C, Chen Y-H, and Wen H-L. 2015. Supporting the annual international black-faced spoonbill census with a low-cost unmanned aerial vehicle. *Ecol Inform* **30**: 170-178

Moreland EE, Cameron MF, Angliss RP, and Peter L. 2015. Evaluation of a ship-based unoccupied aircraft system (UAS) for surveys of spotted and ribbon seals in the Bering Sea pack ice. *J Unmanned Veh Syst* **122**: 114–22.

Mulero-Pázmány M, Negro JJ, and Ferrer M. 2014a. A low cost way for assessing bird risk hazards in power lines: Fixed-wing small unmanned aircraft systems. *J Unmanned Veh Syst* **2**: 5–15.

Mulero-Pázmány M, Stolper R, Essen LD Van, *et al.* 2014b. Remotely piloted aircraft systems as a rhinoceros anti-poaching tool in Africa. *PLoS One* **9**: 1–10.

Pomeroy P, Connor LO, and Davies P. 2015. Assessing use of and reaction to unmanned aerial systems in gray and harbor seals during breeding and molt in the UK. *J Unmanned Veh Syst* **113**: 102–13.

Potapov E, Utekhina I, and Rimlinger D. 2013. Usage of UAV for Surveying Stelle´s Sea Eagle Nests. *Raptors Conserv* **27**: 253–60.

Putzer D. 1989. Wirkung und Wichtung menschlicher Anwesenheit und Störung am Beispiel bestandsbedrohter, an Feuchtgebiete gebundener Vogelarten. *Lanschatspfl u Naturschutz* **29**: 169–94.

Ranftl H. 1990. Auswirkungen des Luftsportes auf die Vogelwelt und die sich daraus ergebenden Forderungen. *Vogel und Luftverkehr* **10**: 24–33.

Ratcliffe N, Guihen D, Robst J, *et al.* 2015. A protocol for the aerial survey of penguin colonies using UAVs. *J Unmanned Veh Syst* **3**: 95–101.

Rümmler M-C, Mustafa O, Maercker J, *et al.* 2015. Measuring the influence of unmanned aerial vehicles on Adélie penguins. *Polar Biol*.

Sweeney KL, Helker VT, Perryman WL, *et al.* 2015. Flying beneath the clouds at the edge of the world: using a hexacopter to supplement abundance surveys of Steller sea lions (*Eumetopias jubatus*) in Alaska. *J Unmanned Veh Syst* **4**: 1–12.

USGS. 2011. Utilizing UAS Raven to estimate sandhill crane abundance. United States.

Vas E, Lescröel A, Duriez O, *et al.* 2015. Approaching birds with drones : first experiments and ethical guidelines. *Biol Lett* **11** (2): 20140754..

Vermeulen C, Lejeune P, Lisein J, *et al.* 2013. Unmanned aerial survey of elephants. *PLoS One* **8**: e54700.

Weissensteiner MH, Poelstra JW, and Wolf JBW. 2015. Low-budget ready-to-fly unmanned aerial vehicles: an effective tool for evaluating the nesting status of canopy-breeding bird species. *J Avian Biol* (46): 425–430
