# Supplementary material for: Unmanned aircraft systems as a new source of disturbance for wildlife: A systematic review
Source: PLoS One. 2017 Jun 21;12(6):e0178448. doi: 10.1371/journal.pone.0178448 (PMC5479521; doi:10.1371/journal.pone.0178448)

**S4 Text: UAS and car sound comparison**

We performed a set of experiments with the aim of comparing the frequencies and intensity of the noise produced by a fixed wing UAS with the noise produced by a car.

**Methods**

Fieldwork was conducted in Doñana Natural Space, (Southwest of Spain) in an open area composed of semi-flooded marsh from mid February to early March in 2010 and 2011 on days with minimal wind. We measured the sound of a fixed wing UAS made from a modified Easy Fly St-330 (for details see Mulero-Pázmány *et al.* 2014b) propelled by an electrical engine and a car Land Rover Defender. We made at least two measurements for each range at different distances: 1, 10, 25, 50, 100, 150, 200 and 250 m, with the aircraft's engine first at full power and then at medium engine power (always hand-hold at 1 m over the ground) and at the same distances with the car engine working at 2500 revolutions per minute.

To study the frequencies emitted by the UAS and the car, we recorded the sound using a Sennheiser E614 microphone (http://en-us.sennheiser.com/instrument-microphone-polarized-condenser-percussion-woodwind-string-e-614) with a frequency response from 40 Hz to 20 kHz and a super-cardioid pattern. Sound characterization was performed downloading the information from the recorder FR-2 Foster into the sonographer Avisoft SasLab Pro (Avisoft Bioacustics. www.avisoft-saslab.com). The background noise analysis showed that at low frequencies it is lower than 80Hz. However, we selected for the analysis the points of the recording where wind background noise is minimal and analyzed a section of the spectra that is six milliseconds. To study the intensity of sound produced by the UAS and the car, we used a hand held sound level meters SLM Cirrus CR: 800C (http://www.cirrusresearch.co.uk/).

Statistical analysis (lineal regression for UAS and car sound intensity) were performed in R version 3.1.1 (R Core Team 2014).

**Results**

The analysis of UAS noise frequencies revealed that the spectrum pattern of energy is high, with the largest amplitude recorded at 1-1.5 kHz, and then it drops to 2 kHz before it levels off at 22 kHz (Figure S1). The effects of increasing distance and reducing engine power are similar, both producing an attenuation of the high frequencies (i.e. at 50 m distance, frequencies over 9.5 KHz disappear). The spectrum pattern of energy of the car is also high and presents similar frequencies to the UAS, with the largest amplitude recorded at 0.2 kHz, and then it falls gradually up to 19 kHz.

As expected, higher frequencies also attenuate when distance increases, but less abruptly than for the UAS (see Figure S2). Regarding sound intensity (figure S3), the Leq dB (A) for UAS ranges between 45 dB (medium power engine) and 70 dB (full power engine) for UAS, compared to about 55 dB for the car, falling down very sharply when increasing distance along the first 50 m and decreasing more smoothly from there. Overall, the UAS at maximum power engine emits more noise intensity than the car, but at medium power the levels are lower than those produced by a car.

**Figures**

Figure S1: UAS Sound frequencies spectrum at full power engine measured at 1 m distance.


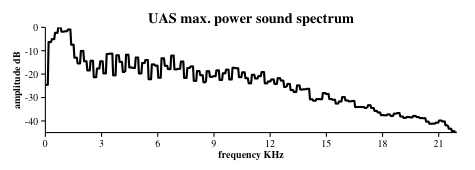


Figure S2: Car sound frequencies spectrum measured at 1 m distance.


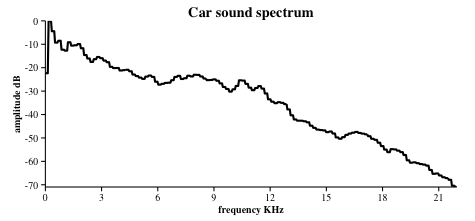


Figure S3: Sound intensity for UAS at medium and maximum power and for a car at different distances. Equations: Leq (UAS med)= 70.27-14.09*log(distance); Leq (car)= 76.41-14.62*log(distance); Leq (UAS max)= 89.88-19.72*log(distance).


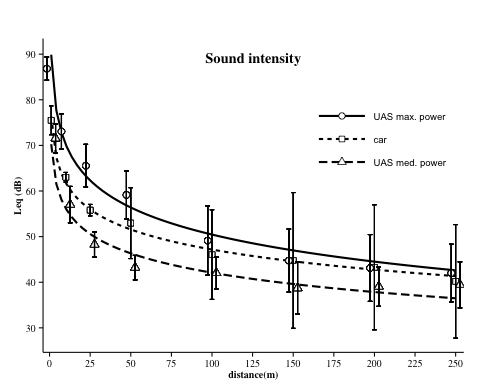

Supplement: S4 Text — (DOCX) [file pone.0178448.s004.docx]
