# Supplementary material for: Unmanned aircraft systems as a new source of disturbance for wildlife: A systematic review
Source: PLoS One. 2017 Jun 21;12(6):e0178448. doi: 10.1371/journal.pone.0178448 (PMC5479521; doi:10.1371/journal.pone.0178448)
Supplement: S5 Text — (DOCX) [file pone.0178448.s005.docx]

**S5 Text: Guidelines to minimize UAS impact on wildlife**

In this annex we provide some guidance that may help UAS users to perform flights minimizing impact on wildlife and can also serve wildlife management authorities to evaluate the potential impact of UAS missions.

There are two basic conditions that must be met in any UAS operation:

1) The UAS has to be reliable and maintained in good conditions. Some spares have to be periodically checked and substituted (e.g. LiPo batteries). Users that personally assemble or modify their platforms should perform tests in controlled non-sensitive areas.

2) The UAS pilot has to be experienced to operate the system safely prior to any ‘real’ application (potentially) involving wildlife. Basic piloting abilities should be guaranteed, either by a certificate issued by the authorities or evaluated with flying maneuvers such as flying in straight lines, making turns, circles, avoiding obstacles, hovering and landing on predetermined sites (see also Kakaes *et al.* 2015 Appendix 2 for a safety pre and post-flight check list).

For UAS flights in sensitive areas such as nature reserves or with the aim of surveying fauna we recommend the following guidelines:

1. Favor silent (electric) or small UAS over noisier (fuel-powered) or larger UAS.
2. Mount the ground control station away from the area of study and in a discrete way, as its presence may disturb the animals. Although the distance depends on the species, 100-300 m may be reasonable for most cases.
3. Conduct missions as short as possible.
4. Perform the flights at the highest altitude possible that still yield useful data. If there is no previous knowledge on the animals’ reaction to UAS, start overflying them at high altitudes (e.g. 300 m AGL) and then gradually descend (e.g. in steps of 50 m) or until you can still retrieve useful data.
5. Avoid gaining altitude, taking off and landing with the UAS above and within 50 m of animals and perform those maneuvers away from the target area.
6. Perform the flight following a series of straight lines favoring the lawn-mower pattern and avoid making turns just above the animals.
7. When possible, minimize flights over sensitive species or during a breeding period.
8. Avoid silhouettes and color patterns that may resemble predator (raptor) shapes and color patterns; favor “long-tailed” (i.e. T-shape) UAS fuselages.
9. If close distance flights (<10 m) from animals are required, avoid direct approaches or harassing the individuals. Approach animals in several consecutive short maneuvers of straight lines, maintaining a constant lateral distance or altitude and preferably avoiding fully vertical approaches.
10. Overflown animals should be surveyed before, during and after UAS flights by an observer independent from the UAS operator. The observer should communicate with the operator and inform the operator about behavioral reactions. In case of undesirable reactions, the operator should increase distance or abort the mission.
11. If the UAS flight purpose is nest inspection, approach the nest when adults are away and at times when unprotected eggs/chicks are safe (e.g. avoid times with unusually high or cold temperatures). During nest approaches, a pilot with good flying abilities is especially relevant, as the risk of possible negative effects on the birds and/or an adult attacking the UAS is increased. If an adult approaches in aggressive attitude (or gets too close), get out of the area as fast as possible.
12. If the flights are performed around raptor territories that previously have shown aggressive behaviors, fly at day times when the temperature is low, such as early morning or late evening because diurnal raptors are less likely to fly in such conditions. But if the raptors are on nest and flee in those time periods, abort the missions because the eggs/chicks are especially sensitive to cold.

Considering the lack of studies about the possible impact that frequent or regular UAS flights may have on wildlife in the medium or long term, we also recommend following the precautionary principle and that environmental authorities carefully assess the pros and contras of UAS flights, particularly in sensitive areas, and only authorize flight proposals that are well justified.

These guidelines have been suggested based on the available UAS impact data and personal experience with UAS wildlife overflights of the authors. We recommend conducting more research on UAS impact on wildlife and that the guidelines are periodically reviewed when more information is available.

**Literature cited**

Kakaes K, Greenwood F, Lippincot M, *et al.* 2015. Drones and aerial observation: new technologies for property rights, human rights, and global development. A primer. In: New America. Creative commons.
