# Supplementary material for: Unmanned aircraft systems as a new source of disturbance for wildlife: A systematic review
Source: PLoS One. 2017 Jun 21;12(6):e0178448. doi: 10.1371/journal.pone.0178448 (PMC5479521; doi:10.1371/journal.pone.0178448)
Supplement: S1 Table — (DOCX) [file pone.0178448.s006.docx]

**S1 Table**: Results of the GLMMs exploring the factors affecting the probability of observing a reaction in wildlife exposed to UAS flights (Model 1) and the determinants of the probability of observing an active vs. a passive response to the UAS (Model 2). Two asterisk (**) represent significant results to the standard level (i.e. 95% Bayesian credible intervals (CrI) not including zero) and one asterisk (*) indicate results close to standard significance levels (i.e. 90% CrI does not include zero).

|  |  |  | **Model 1:**  **Probability of observing impact (N=167**§**)** | |  | **Model 2:**  **Probability of the response being active (N= 106**§**)** | |
| --- | --- | --- | --- | --- | --- | --- | --- |
| Effects | |  | Estimate | CrI 95% |  | Estimate | CrI 95% |
| Intercept | |  | 8.63 | -0.61; 17.78 |  | 4.12 | 0.91; 7.38 |
| Flight pattern | |  |  |  |  |  |  |
|  | close up |  | / | / |  | / | / |
|  | lawn-mower |  | -8.78* | -18.03; 0.09 |  | -2.01 | -5.26; 1.07 |
|  | hobby |  | -6.49 | -18.96; 6.56 |  | -1.15 | -6.88; 4.53 |
| Engine type | |  |  |  |  |  |  |
|  | electric |  | / | / |  | / | / |
|  | fuel |  | 5.66* | -0.77; 12.21 |  | 1.10 | -2.94; 5.10 |
| Animal type | |  |  |  |  |  |  |
|  | large birds |  | / | / |  | / | / |
|  | smaller birds |  | -1.51 | -3.57; 0.56 |  | -0.24 | -1.87; 1.4 |
|  | flightless birds |  | 1.39 | -7.23; 9.73 |  | -2.06 | -5.2; 1.14 |
|  | terrestrial mammals | | -3.52** | -6.30; -0.78 |  | -3.61 | -6.47; -0.68** |
|  | underwater species | | -14.03** | -24.57; -3.38 |  | *na* | *na* |
| *Models 1: response variable = 0 (no observed wildlife response) vs. 1 (alert or active response observed); binomial distribution (link = logit); random factors = reference + family + species.*  *Models 2: response variable = 0 (weaker response: alert reaction) vs. 1 (stronger active response: escape or attack); binomial distribution (link = logit); random factors = reference + family + species.*  *“/” represents the category of reference to which the others are contrasted and “na” means not applicable because no impact was found in these species.*  *§Note that these represent total raw sample sizes. However, in mixed models the effective sample sizes are smaller lying somewhere between the total sample sizes and the number of clusters determined by the random factors.* | | | | | | | |
|  | | | | | | | |
