# Supplementary material for: Unmanned aircraft systems as a new source of disturbance for wildlife: A systematic review
Source: PLoS One. 2017 Jun 21;12(6):e0178448. doi: 10.1371/journal.pone.0178448 (PMC5479521; doi:10.1371/journal.pone.0178448)
Supplement: S2 Table — (DOCX) [file pone.0178448.s007.docx]

**S2 Table**: Results of the GLMMs investigating the factors determining the AGL (Model 3 and 4) or distance to the fauna (Model 5) at which UAS were when an active response (mostly escape) was observed. Two asterisk (**) indicate significant results to the standard level (i.e. 95% Bayesian credible intervals (CrI) not including zero) and one asterisk (*) represent results near standard significance levels (i.e. 90% CrI does not include zero).

|  | |  | **Model 3:**  **AGL at which an active response was observed during "target-oriented" flights (N= 12**§**)** | |  | **Model 4:**  **AGL at which an active response was observed during "Lawn-mower" flights**  **(N= 27**§**)** | |  | **Model 5:**  **Distance at which the UAV was from animals when active response was observed during "Hobby" flights (N= 21**§**)** | |
| --- | --- | --- | --- | --- | --- | --- | --- | --- | --- | --- |
| Effects | |  | Estimate | BCI 95% |  | Estimate | BCI 95% |  | Estimate | BCI 95% |
| Intercept | |  | 0.69 | -0.21; 1.60 |  | 3.34 | 2.11; 4.56 |  | 5.44 | 4.46; 6.41 |
| Life history stage | |  |  |  |  |  |  |  |  |  |
|  | breeding |  | / | / |  | / | / |  | / | / |
|  | non breeding |  | 0.43** | 0.09; 0.76 |  | 0.78** | 0.09; 1.48 |  | 0.55** | 0.14; 0.97 |
| UAV size | |  | 0.003** | 0.002; 0.004 |  | 0.0002* | -0.0006; 0.0009 |  |  |  |
| Engine type | |  |  |  |  |  |  |  |  |  |
|  | electric |  |  |  |  | / | / |  |  |  |
|  | fuel |  |  |  |  | 0.39 | -0.72; 1.48 |  |  |  |
| Level of aggregation | | |  |  |  |  |  |  |  |  |
|  | large group |  |  |  |  |  |  |  | / | / |
|  | medium group |  |  |  |  |  |  |  | -0.23 | -0.75; 0.32 |
|  | small groups |  |  |  |  |  |  |  | -0.42** | -0.80; -0.07 |
|  | solitary |  |  |  |  |  |  |  | -1.24** | -2.32; -0.21 |
| *Models 3 and 4: response variable = log (AGL); normal distribution (link = identity); random factors = reference + family + species.*  *Models 5: response variable = log (escape distance); normal distribution (link = identity); random factors = reference + family + species.*  *“/”: represents the category of reference to which the others are contrasted.*  *§Note that these represent total raw sample sizes. However, in mixed models the effective sample sizes are smaller lying somewhere between the total sample sizes and the number of clusters determined by the random factors.* | | | | | | | | | | |
