# Supplementary figures and images for: Unmanned aircraft systems as a new source of disturbance for wildlife: A systematic review
Source: PLoS One. 2017 Jun 21;12(6):e0178448. doi: 10.1371/journal.pone.0178448 (PMC5479521; doi:10.1371/journal.pone.0178448)

# PRISMA 2009 Flow Diagram

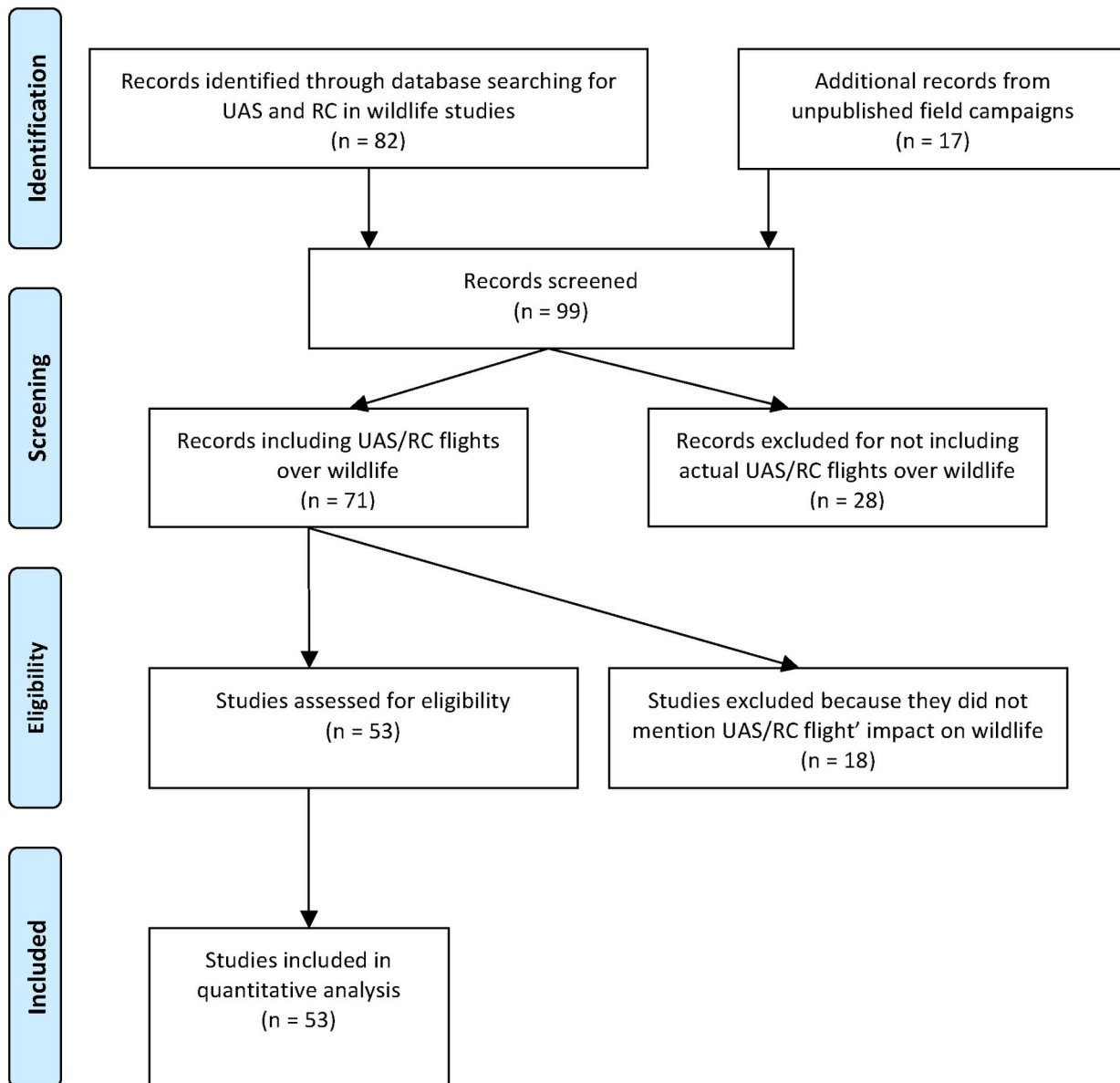

Supplement: S1 Checklist — (PDF) [file pone.0178448.s008.pdf]
